# Rationing tests for drug-resistant tuberculosis – who are we prepared to miss?

**DOI:** 10.1186/s12916-016-0576-8

**Published:** 2016-03-23

**Authors:** Laura J. Martin, Martha H. Roper, Louis Grandjean, Robert H. Gilman, Jorge Coronel, Luz Caviedes, Jon S. Friedland, David A. J. Moore

**Affiliations:** Laboratorio de Investigación de Enfermedades Infecciosas, Universidad Peruana Cayetano Heredia, San Martín de Porras, Lima Peru; Section of Infectious Diseases & Immunity & Wellcome Trust Imperial College Centre for Clinical Tropical Medicine, Imperial College London, London, UK; LSHTM TB Centre and Department of Clinical Research, London School of Hygiene and Tropical Medicine, London, UK; Department of International Health, Johns Hopkins Bloomberg School of Public Health, Baltimore, Maryland USA

**Keywords:** Microscopic-observation drug-susceptibility assay, Multidrug-resistant tuberculosis, Tuberculosis, Drug Susceptibility Testing

## Abstract

**Background:**

Early identification of patients with drug-resistant tuberculosis (DR-TB) increases the likelihood of treatment success and interrupts transmission. Resource-constrained settings use risk profiling to ration the use of drug susceptibility testing (DST). Nevertheless, no studies have yet quantified how many patients with DR-TB this strategy will miss.

**Methods:**

A total of 1,545 subjects, who presented to Lima health centres with possible TB symptoms, completed a clinic-epidemiological questionnaire and provided sputum samples for TB culture and DST. The proportion of drug resistance in this population was calculated and the data was analysed to demonstrate the effect of rationing tests to patients with multidrug-resistant TB (MDR-TB) risk factors on the number of tests needed and corresponding proportion of missed patients with DR-TB.

**Results:**

Overall, 147/1,545 (9.5 %) subjects had culture-positive TB, of which 32 (21.8 %) had DR-TB (MDR, 13.6 %; isoniazid mono-resistant, 7.5 %; rifampicin mono-resistant, 0.7 %). A total of 553 subjects (35.8 %) reported one or more MDR-TB risk factors; of these, 506 (91.5 %; 95 % CI, 88.9–93.7 %) did not have TB, 32/553 (5.8 %; 95 % CI, 3.4–8.1 %) had drug-susceptible TB, and only 15/553 (2.7 %; 95 % CI, 1.5–4.4 %) had DR-TB. Rationing DST to those with an MDR-TB risk factor would have missed more than half of the DR-TB population (17/32, 53.2 %; 95 % CI, 34.7–70.9).

**Conclusions:**

Rationing DST based on known MDR-TB risk factors misses an unacceptable proportion of patients with drug-resistance in settings with ongoing DR-TB transmission. Investment in diagnostic services to allow universal DST for people with presumptive TB should be a high priority.

## Background

Among notified patients with pulmonary tuberculosis (TB) in 2014, the estimated total number of cases of multidrug-resistant TB (MDR-TB) worldwide was 480,000, of which only approximately 123,000 were detected [1]. The known MDR-TB epidemic represents the tip of a dangerous iceberg, with unidentified patients going on to develop advanced disease that is difficult and expensive to treat, whilst at the same time transmitting MDR-TB infection to their contacts and threatening recent progress in global TB control.

Successful TB control programmes invite large numbers of individuals with non-specific symptoms, including cough, fever or weight loss, to attend their local health facility for assessment to exclude TB. In the case of MDR-TB disease, treatment outcomes are best and most cost effective when patients receive drug susceptibility testing (DST) at diagnosis [2]. However, providing baseline DST alongside TB diagnosis to all symptomatic individuals represents a huge undertaking and many countries lack the capacity to test all patients [1].

New diagnostic tests have been developed, include Xpert MTB/RIF, which detects rifampicin resistance as a marker for MDR-TB. This test is endorsed by the World Health Organization (WHO) and has been implemented in many resource-poor settings worldwide [1]. However, the relatively high cost of the test means that, in settings with constrained resources, it is suggested that only individuals with MDR-TB risk factors are selected for testing. The WHO Xpert implementation policy also suggests that Xpert DST should be applied as the initial diagnostic test in place of conventional microscopy, culture and DST in patients suspected of having MDR-TB or HIV-associated TB [1].

This study was based in Lima, Peru, where MDR-TB represents 3.9 % and 35 % of new and re-treatment TB cases, respectively. In the course of a diagnostic evaluation study [3], we collected detailed clinical, epidemiological, microbiological and socioeconomic data from patients who presented to government health centres for TB investigations. This analysis investigates the hypothesis that risk stratification to reduce the number of DSTs performed results in a major failure in case detection of drug-resistant TB (DR-TB).

## Method

### Study design, participants and settings

The study site, design and participants have been reported previously [3]. Patients aged ≥18 years presenting with respiratory symptoms and attending participating TB clinics for the evaluation of possible TB were recruited to the study after providing written informed consent. At presentation, patients’ responses to a questionnaire were documented and two samples of sputum were submitted to the National TB Programme for routine Ziehl–Neelsen smear followed by culture and DST.

### Laboratory methods

#### Detection of *Mycobacterium tuberculosis* (MTB)

Sputum samples were decontaminated according to the NaOH-NALC method [4]. Following this, an aliquot was used for microscopic examination of auramine-stained sputum smears and the remainder underwent parallel TB culture on Löwenstein–Jensen medium and in MODS liquid medium.

#### DST for rifampicin and isoniazid

Direct DST was performed with the MODS assay [5, 6]. Indirect DST was performed using the proportion method [7] for isolates from the Löwenstein–Jensen culture (by an external laboratory) and with the automated MBBacT system [8] for isolates from the automated mycobacterial culture.

### Field methods

Clinical, epidemiological and socioeconomic data were collected using a standardized nurse-administered questionnaire. Data recorded included (1) the presence and duration of symptoms; cough, expectoration, fever, breathlessness, haemoptysis, weight loss, night sweats and chest pain; (2) risk factor exposures for TB or DR-TB, HIV infection, prior TB treatment, known TB or MDR-TB contact, healthcare or prison service worker, recent hospitalization or prison incarceration, drug and alcohol use, or BCG vaccination; and (3) indicators of household wealth related to income, education, overcrowding and sanitation contributing to the “Necesidades Basicas Insatisfechas”, a locally validated poverty score described and used by the Economic Commission for Latin America and the Caribbean [9].

### Definitions

DR-TB was defined as strains of MTB resistant to rifampicin and/or isoniazid. Isoniazid mono-resistance was defined as resistance to isoniazid by any method without accompanying rifampicin resistance, rifampicin mono-resistance was defined as resistance to rifampicin by any method without accompanying isoniazid resistance, and multidrug resistance was defined as resistance to both rifampicin and isoniazid by any method [1]. The remaining strains were defined as drug susceptible.

MDR-TB risk factors included previous TB treatment, contact with a known MDR-TB patient, malabsorption, exposure to environments with high rates of MDR-TB (healthcare workers, prison workers and residents, previous hospitalisation) and HIV infection [10, 11].

### Statistical analysis

Data were analysed using Stata 11 (College Station, TX: StataCorp LP 2010). The principal outcomes of interest were the proportion of DR-TB diagnosed among the population of non-selected subjects and the proportion of DR-TB diagnosed in subgroups of selected subjects with and without MDR-TB risk factors. Subgroups considered were subjects with previous TB, contact with MDR-TB, health workers, prison workers or residents, and HIV infection (MDR-TB risk factors [10]), by all subjects with any MDR-TB risk factor, and by smear positive subjects only. Analyses were also performed with and without inclusion of sputum smear results to reflect the recommendation that rapid testing should be used as the initial diagnostic test in adults with risk factors for MDR-TB in place of an initial sputum smear microscopy [1, 12]. The inclusion of the smear microscopy result evaluated the sensitivity of using DST as a follow-on test after initial smear microscopy.

The following calculations were undertaken for each subgroup in which subjects would be selected for DST: (1) number of DSTs that would be performed, (2) number and proportion of DR-TB patients diagnosed, and (3) number and proportion of DR-TB patients that would be missed.

The number of subjects in each MDR-TB risk factor subgroup were calculated to demonstrate the reduction in burden of DSTs required should testing be restricted to these subgroups.

### Ethics review

Study protocol and consent forms were approved by the institutional review boards of Universidad Peruana Cayetano Heredia, Asociación Benéfica PRISMA, Dirección de Salud–III Lima Norte and IV Lima Este, Johns Hopkins Bloomberg School of Public Health, and Imperial College London.

## Results

### Population characteristics

Pre-treatment sputum samples were provided by 1,545 patients who had presented for TB testing (40.2 % male, mean age 36.1 years), of which 147 (9.5 %) were culture positive for TB (50.3 % smear positive, Table 1). Drug susceptibility testing identified 115 strains susceptible to rifampicin and isoniazid (7.4 % of all samples and 78.2 % of culture positive samples), 20 MDR strains (1.3 % of all samples, 13.6 % of culture positive samples), 11 isoniazid mono-resistant strains (0.7 % of all samples, 7.5 % of culture positive samples), and one rifampicin mono-resistant strain (0.1 % of all samples, 0.7 % of culture positive samples). Overall, 520 subjects (33.7 %) met criteria for living in poverty and 250 (16.2 %) for overcrowding.

**Table 1:** Study participant demographic, socioeconomic, epidemiological and clinical characteristics

	All patients				Sputum culture negative for TB			Sputum culture positive for TB											
								Drug-susceptible TB^a^				MDR-TB				Mono-resistant TB^a^			
	N			Unknown (n)	n		Unknown (n)	n			Unknown (n)	n			Unknown (n)	n			Unknown (n)
		Percentage of all subjects	Percentage of all patients			Percentage of all subjects			Percentage of all subjects	Percentage of all patients with drug sensitive TB			Percentage of all subjects	Percentage of patients with MDR-TB			Percentage of all subjects	Percentage of patients with mono-resistant TB	
Number of subjects	1,545	100.0			1,398	90.5	0	115	7.4	100.0	0	20	1.3	100.0	0	12	0.8	100.0	0
Age (mean)	36			0	37		0	32			0	32			0	32			0
Male sex	621	40.2	49.0	0	549	35.5	0	56	3.6	48.7	0	9	0.6	45.0	0	7	0.5	58.3	0
Smear positive	82	5.3	50.3	0	8	0.5	0	60	3.9	52.2	0	11	0.7	55.0	0	3	0.2	25.0	0
Poverty^b^	520	33.7	28.6	33	478	30.9	27	34	2.2	29.6	5	6	0.4	30.0	1	2	0.1	16.7	0
Overcrowding^c^	250	16.2	15.0	20	228	14.8	16	14	0.9	12.2	4	7	0.5	35.0	0	1	0.1	8.3	0
TB contact	809	52.4	51.0	4	734	47.5	4	59	3.8	51.3	0	11	0.7	55.0	0	5	0.3	41.7	0
Smoker (current/ex)	89	5.8	18.4	7	62	4.0	4	26	1.7	22.6	3	1	0.1	5.0	0	0	0.0	0.0	0
Drug user	23	1.5	4.1	3	17	1.1	3	6	0.4	5.2	0	0	0.0	0.0	0	0	0.0	0.0	0
Excess alcohol	185	12.0	19.7	3	156	10.1	3	25	1.6	21.7	0	3	0.2	15.0	0	1	0.1	8.3	0
Clinical information																			
Prior BCG vaccine	1,338	86.6	90.5	21	1,205	78.0	21	105	6.8	91.3	0	18	1.2	90.0	0	10	0.6	83.3	0
Cough	1,290	83.5	87.1	1	1,162	75.2	1	101	6.5	87.8	0	17	1.1	85.0	0	10	0.6	83.3	0
Fever	514	33.3	47.6	5	444	28.7	4	50	3.2	43.5	1	14	0.9	70.0	0	6	0.4	50.0	0
Haemoptysis	238	15.4	31.3	5	192	12.4	5	41	2.7	35.7	0	3	0.2	15.0	0	2	0.1	16.7	0
Weight loss	754	48.8	69.4	8	652	42.2	8	80	5.2	69.6	0	15	1.0	75.0	0	7	0.5	58.3	0
Night sweats	567	36.7	49.0	8	495	32.0	7	54	3.5	47.0	1	12	0.8	60.0	0	6	0.4	50.0	0
Chest pain	940	60.8	66.7	7	842	54.5	7	77	5.0	67.0	0	12	0.8	60.0	0	9	0.6	75.0	0
Breathlessness	680	44.0	51.0	13	605	39.2	13	55	3.6	47.8	0	12	0.8	60.0	0	8	0.5	66.7	0

^a^ Susceptible to isoniazid and rifampicin; 11 isoniazid mono-resistant, 1 rifampicin mono-resistant

^b^ Participants with a socioeconomic score indicating poverty [9]

^c^ Participants who meet criteria for overcrowding [9] if total household residents/number bedrooms >3.4

TB, Tuberculosis; MDR-TB, Multidrug-resistant tuberculosis

### Factors associated with DR-TB diagnosis amongst participants presenting for TB testing

Among the 1,545 participants, 553 (35.8 %) patients had an MDR-TB risk factor (Table 2). Within this group, 47 (8.5 %) were sputum culture-positive for TB, 32 (2.1 %) had TB sensitive to rifampicin and isoniazid, 11 (0.7 %) had MDR-TB, four (0.3 %) had mono-resistant TB, 506 (91.5 %) were sputum culture-negative for TB, and 32 (2.1 %) had TB susceptible to rifampicin and isoniazid.

**Table 2:** Study participant risk factors and microscopy and culture/drug-susceptible tuberculosis results

	All subjects	Sputum culture negative for TB			Sputum culture positive for TB											
					Drug-susceptible TB				MDR-TB				Mono-resistant TB			
				Unknown (n)	n			Unknown (n)	n			Unknown (n)	n			Unknown (n)
	n	n	Percentage of all subjects			Percentage of all subjects	Percentage of all drug sensitive TB patients			Percentage of all subjects	Percentage of all MDR-TB patients			Percentage of all subjects	Percentage of all mono-resistant TB patients	
All patients	1,545	1,398	90.5	0	115	7.4	100	0	20	1.3	100	0	12	0.8	100	0
≥1 MDR-TB-TB risk factor^a^	553	506	32.8	0	32	2.1	27.8	0	11	0.7	55.0	0	4	0.3	33.3	0
≥1 MDR-TB risk factor or smear Positive sputum^b^	604	509	32.9	0	74	4.8	64.3	0	16	1.0	80.0	0	5	0.3	41.7	0
Previous TB	309	278	18.0	4	20	1.3	17.4	0	8	0.5	40.0	0	3	0.2	25.0	0
Known contact with MDR TB	161	153	9.9	8	3	0.2	2.6	0	3	0.2	15.0	0	2	0.1	16.7	0
Health worker	66	62	4.0	4	3	0.2	2.6	0	1	0.1	5.0	0	0	0.0	0.0	0
Current/ex prison worker or resident	38	32	2.1	4	5	0.3	4.3	0	1	0.1	5.0	0	0	0.0	0.0	0
HIV infection	18	18	1.2	4	0	0.0	0.0	1	0	0.0	0.0	0	0	0.0	0.0	0

^a^ MDR-TB risk factors as recommended by the WHO [10]; Previous TB treatment, Contact with known MDR-TBpatient), exposure to environments with high rates MDR TB (health care workers, prison workers & residents, previous hospitalisation), HIV infection

^b^Represents DST use as a follow on test after sputum microscopy

TB, Tuberculosis; MDR-TB, Multidrug-resistant tuberculosis

In total, 809 (52.3 %) patients reported prior TB contact and 734 of these patients (90.7 %) had a negative sputum culture; 161 subjects reported MDR-TB contact (10.4 %) of whom 153 (95.0 %) had a negative sputum culture, three had TB sensitive to rifampicin and isoniazid, three had MDR-TB, and two had mono-resistant TB. One fifth of patients (n = 309) were previously treated; 278 (90.0 %) were TB culture-negative and 12 (3.9 %) were diagnosed with DR-TB, representing 37.5 % of DR-TB in this unselected study population. Health workers accounted for 4.3 % of the population (n = 66), and current or ex-prison workers/residents made up 2.5 % (n = 38). One subject in each of these groups was diagnosed with DR-TB.

### The impact of DST rationing upon numbers of patients tested and DR-TB cases identified

If only those patients with a MDR-TB risk factor had been selected for DST at diagnosis, 46.9 % (95 % CI, 29.6–64.2 %; n = 15) of patients with DR-TB (n = 32) would have been detected.

**Fig. 1:**
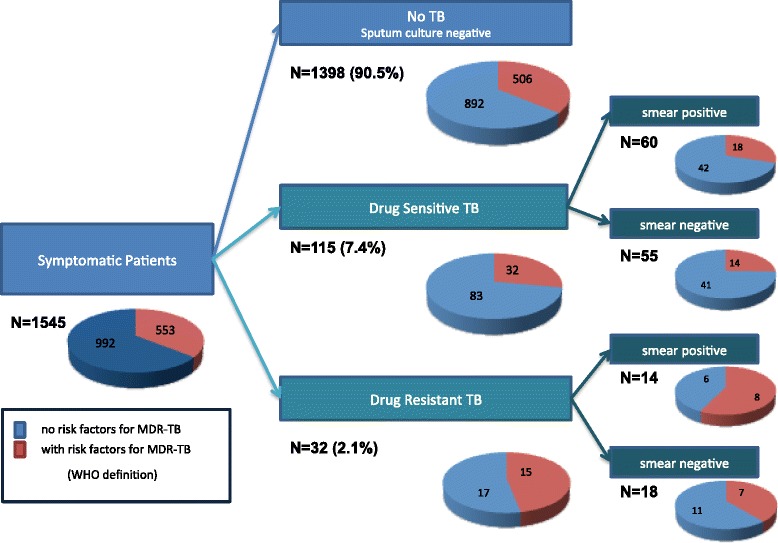
Symptomatic patients with multidrug-resistant tuberculosis risk factors for each group

Within the group of patients with DR-TB (n = 32), 43.8 % (95 % CI, 26.6–60.9 %; n = 14) were smear positive; 42.9 % (five MDR-TB and one isoniazid mono-resistant TB) of these smear positive patients with DR-TB did not have any MDR-TB risk factors (Fig. 1).

**Fig. 2:**
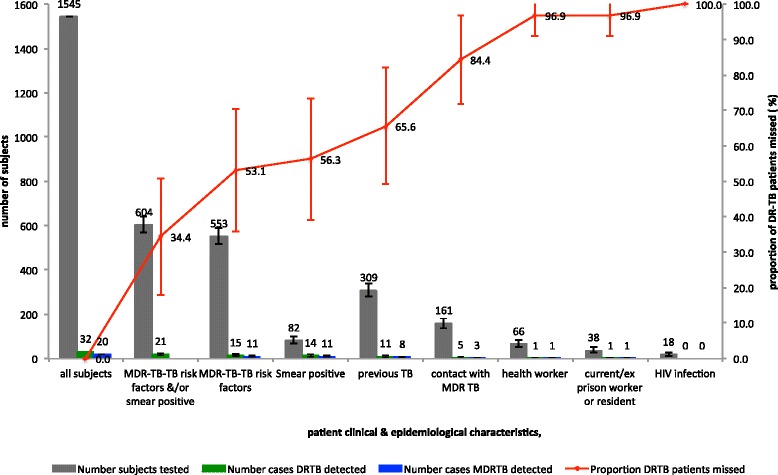
Percentages of drug-resistant tuberculosis patients detected by testing strategies

Overall, 28 % (32/115) of patients with DR-TB had a risk factor for MDR-TB whilst 36 % (506/1,398) of patients who presented for TB testing but did not have TB reported a risk factor for MDR-TB (Fig. 1).

If MDR-TB risk factors alone were used as criteria for a DST at diagnosis, this would lead to 35.8 % (553/1,545; 95 % CI, 33.4–38.2 %) of patients being tested and 46.9 % (15/32; 95 % CI, 29.6–64.2 %) of DR-TB cases being detected; 64.2 % (992/1,545) of cases would not meet the criteria for testing at the cost of missing 53.1 % (17/32; 95 % CI, 35.8–70.4 %) of DR-TB cases (Fig. 2, Table 3).

If MDR-TB risk factors and/or sputum smear positivity together were used as criteria for a DST at diagnosis, this would lead to 39.1 % (604/1,545; 95 % CI, 36.7–41.5 %) of cases being tested and 65.6 % (21/32; 95 % CI, 49.2–82.1) of DR-TB cases being detected; 60.9 % (941/1,545; 95 % CI, 58.5–63.3 %) would not meet the criteria for testing at the cost of missing 34.4 % (11/32; 95 % CI, 17.9–50.8 %; Fig. 2, Table 3) of DR-TB cases.

**Table 3:** Performance of drug-susceptibility testing with risk stratification for detection of drug-resistant tuberculosis (DR-TB)

	N	Percentage of subjects tested if only this subgroup was selected for drug-susceptibility testing (95 % CI)	DR-TB cases identified from subgroup (n)	Percentage of DR-TB cases identified (95 % CI)	DR-TB cases missed (n)	Percentage of DR-TB cases missed (95 % CI)	Numbers needed to test to identify one DR-TB case
All patients	1,545	100.0	32	100.0	0	0.0	48
Patient subgroups							
≥1 MDR-TB risk factor^a^	553	35.8 (33.4–38.2)	15	46.9 (29.6–64.2)	17	53.1 (35.8–70.4)	37
≥1 MDR-TB risk factor or smear positive sputum^b^	604	39.1 (36.7–41.5)	21	65.6 (49.2–82.1)	11	34.4 (17.9–50.8)	29
Sputum smear positive	82	5.3 (4.2–6.4)	14	43.8 (26.6–60.9)	18	56.3 (39.1–73.4)	6
Previous TB	309	20.0 (18.0–22.0)	11	34.4 (17.9–50.8)	21	65.6 (49.2–97.0)	28
Known contact with MDR-TB	161	10.4 (8.9–11.9)	5	15.6 (3.0–28.2)	27	84.4 (71.8–97.0)	32
Health worker	66	4.3 (3.3–5.3)	1	3.1 (0–9.2)	31	96.9 (90.8–100.0)	66
Current/ex prison worker or resident	38	2.5 (1.7–3.2)	1	3.1 (0–9.2)	31	96.9 (90.8–100.0)	38
HIV infection	18	1.2 (0.6–107)	0	0	32	100.0	n/a

DR-TB, Drug-resistant tuberculosis; MDR-TB, Multidrug-resistant tuberculosis; CI, Confidence interval

If all patients were tested and all DR-TB cases identified, then 48 tests per DR-TB case identified would be performed (Fig. 2, Table 3). This compares to 37 tests per DR-TB case identified if MDR-TB risk factors alone were used (albeit at a cost of 53.1 % of DR-TB cases missed) and 29 tests per DR-TB case identified would be performed if risk factors and/or smear positivity were used (at a cost of 34.4 % missed DR-TB cases).

## Discussion

The key finding of this work is that epidemiological risk stratification to target DST testing and reduce testing burden comes at too great a cost in MDR-TB cases overlooked, regardless of the approach used.

Patient selection using WHO-defined risk factors for MDR-TB [10] would consign 53.1 % (17/32) of patients to a missed diagnosis of DR-TB in this population. Even if patients with smear-positive sputum were also included in a testing strategy, 34.4 % (11/32) of DR-TB cases would be missed.

This study suggests that epidemiological risk factors for MDR-TB are neither sensitive nor specific for the identification of patients with MDR or mono-resistant TB; instead, MDR-TB risk factors are distributed among all individuals that present for testing. Only 46.9 % of patients diagnosed with DR-TB had a risk factor for MDR-TB and a significant proportion of patients with DR-TB or without culture-positive TB (27.8 % and 36.2 %, respectively) described a risk factor for MDR-TB when they presented to the health centre.

There are strikingly low rates of MDR-TB disease among ‘high risk’ groups despite this study recruiting an unselected group of patients that attended for TB testing in a TB endemic area. The large groups of current or ex-healthcare workers and current or ex-prisoners or prison workers yielded only a single patient with MDR-TB in each group. Among the smear-positive patients who were diagnosed with DR-TB disease, 42.9 % (6/14) did not have a risk factor for MDR-TB. This group of infectious smear-positive patients, five with MDR-TB and one with isoniazid mono-resistant disease, would have missed DST testing at presentation if only patients with MDR-TB risk factors were selected for testing without an initial sputum smear. This is a sobering finding since, by the end of 2014, 69 % of countries reported following national guidelines that recommend patients with risk factors for MDR-TB undergo Xpert MTB/RIF as the initial diagnostic investigation [1, 12].

The repercussions of missed opportunities for early diagnosis of active DR-TB are clear; unidentified DR-TB patients progress to develop more severe disease, requiring more prolonged and expensive treatment and have worse outcomes than if the disease was correctly identified and treated at presentation [2, 13–16]. Unidentified patients with DR-TB are more likely to infect contacts than those on appropriate treatment. Viewed from an economic perspective, this generates additional treatment costs, estimated to lie within the range US$ 5,000–10,000 per person with MDR-TB [1, 17, 18]. If drug resistance were to progress to extensively DR-TB in patients with unidentified and untreated DR-TB, the estimated cost of treatment rises to US$ 26,392 per person [19]. These costs are likely to exceed funds saved by rationing initial DSTs to only those patients with MDR-TB risk factors. This study demonstrates that using universal rather than selective testing would have led to an increase in initial DST numbers required to 37–48 per DR-TB case detected, a comparatively small expenditure compared to costs of treating missed DR-TB patients.

A major strength of this study was its implementation under real life conditions, recruiting a large number of unselected patients who self-reported to government TB program health centres and were able to provide detailed clinical and socioeconomic information. The information required to ascertain whether a patient would fulfil WHO risk factors for MDR-TB criteria was obtained for almost all patients. Patients were not asked specifically about previous TB treatment in the private sector or previous use of TB drugs of poor or unknown quality, as is detailed in the WHO risk factor criteria; however, these patients were likely to form a subset of the previously treated patients who were included as being at risk of MDR-TB. We did not ask specifically about diseases of malabsorption in this population, a pre-existing condition that is considered to be a risk factor for MDR-TB, though the attributable importance of this is likely to be negligible. Arguably, a study limitation is that the 147 patients with TB (including 32 with DR-TB) constitute a small proportion of the 1,545 individuals tested in this study. However, it is precisely the scale of these proportions that presents a real challenge for health systems required to identify relatively small numbers of TB patients in large populations of symptomatic individuals. The aim of targeting DST at diagnosis is to identify patients with DR-TB who would benefit from individualised treatment. Patients with mono-resistant disease were included in the analysis for this reason. Mono-resistant disease risk factors distinct from MDR-TB risk factors have not been identified, but are clinically likely to overlap. The study was performed at a single site but data are likely to be generalizable to settings where MDR-TB transmission is established and MDR-TB is not still restricted to patients with relapse [20].

The analysis reinforces findings from previous studies that patient risk factors are unreliable for predicting who will benefit from DST at diagnosis [21–25]. It raises important questions about true cost savings when DST is limited to a relatively small subset of patients presenting with possible TB symptoms.

Cost analyses have compared the Xpert MTB/RIF test to conventional culture followed by DST [26, 27], but not to rapid phenotypic culture methods [6, 28–30] that could be sufficiently inexpensive to permit testing of all patients presenting with suspected TB; therefore, a detailed economic evaluation that includes these methods would be valuable future work.

Preferential investment in laboratory infrastructure would permit the provision of affordable universal DST at diagnosis, as well as providing facilities for confirmatory diagnostic testing, monitoring throughout treatment and the development of second-line drug resistance profiles for individualised treatment regimes. Laboratory strengthening to provide these important tools is an essential process so that a high standard of care can be provided to all of the individuals that require it.

## Conclusions

Where resources are constrained, TB programmes are forced to make choices about which patients should undergo DST, despite the call from the WHO End TB strategy for universal testing [31]. The unspoken question is not “who can we afford to test?”, but rather “who can we afford to not test?” The argument for universal DST for TB patients is not complex: (1) early diagnosis (and effective treatment) of DR-TB reduces morbidity and interrupts transmission, (2) the cost of treating DR-TB is extremely high, (3) the costs averted by preventing secondary cases through earlier recognition and diagnosis of DR-TB are likely to outweigh the costs of testing, (4) MDR-TB risk stratification is a blunt and inaccurate tool, and (5) as shown here, the trade-off of targeted testing is a systematic failure to detect all DR-TB for a relatively trivial reduction in testing volume. Diagnostic tests, such as Xpert MTB/RIF [12], Genotype MTB/DR plus [32], MODS [3, 5] and the nitrate reductase assay [28], which deliver direct DST at the same time as making the TB diagnosis, go beyond asking “which TB patients should be tested?” to “which patients with presumptive TB should be tested?” If the goal of global elimination is genuinely intended, surely the only answer is “all”.

## References

[1] World Health Organization (2015). Global tuberculosis report.

[2] Oxlade O, Falzon D, Menzies D (2012). The impact and cost-effectiveness of strategies to detect drug resistant tuberculosis. Eur Respir J.

[3] Moore DAJ, Evans CAW, Gilman RH, Caviedes L, Coronel J, Vivar A (2006). Microscopic-observation drug-susceptibility assay for the diagnosis of TB. N Engl J Med..

[4] Laboratory Services in TB Control. Parts I, II, and III. Publication No. WHO/tb/98.258. Geneva: WHO; 1998

[5] Caviedes L, Moore DAJ (2007). Introducing MODS: a low-cost, low-tech tool for high-performance detection of tuberculosis and multidrug resistant tuberculosis. Indian J Med Microbiol..

[6] Moore DAJ, Mendoza D, Gilman RH, Evans CAW, Hollm Delgado M-G, Guerra J (2004). Microscopic observation drug susceptibility assay, a rapid, reliable diagnostic test for multidrug-resistant tuberculosis suitable for use in resource-poor settings. J Clin Microbiol..

[7] Kent PT, Kubica GP (1985). Public Health Mycobacteriology: A Guide for the Level III laboratory. CDC 86-216547.

[8] Tortoli E, Mattei R, Savarino A, Bartolini L, Beer J (2000). Comparison of Mycobacterium tuberculosis susceptibility testing performed with BACTEC 460 TB (Becton Dickinson) and MB/BacT (Organon Teknika) systems. Diagn Microbiol Infect Dis..

[9] Feres JC, Mancero X (2001). El método de las necesidades básicas insatisfechas (NBI) y sus aplicaciones en América Latina.

[10] World Health Organization (2008). Guidelines for the programmatic management of drug-resistant tuberculosis 2008.

[11] World Health Organization (2014). Companion handbook to the WHO guidelines for the programmatic management of drug-resistant tuberculosis.

[12] World Health Organization. Xpert MTB/RIF Assay for the Diagnosis of Pulmonary and Extrapulmonary TB in Adults and Children Policy Update. 2013. http://www.who.int/tb/publications/xpert-mtb-rif-assay-diagnosis-policy-update/en/. Accessed 9 November 2015.

[13] Corbett EL, Bandason T, Duong T, Dauya E, Makamure B, Churchyard GJ (2010). Comparison of two active case-finding strategies for community-based diagnosis of symptomatic smear-positive tuberculosis and control of infectious tuberculosis in Harare, Zimbabwe (DETECTB): a cluster-randomised trial. Lancet..

[14] Churchyard GJ, Fielding K, Roux S, Corbett EL, Chaisson RE, De Cock KM (2011). Twelve-monthly versus six-monthly radiological screening for active case-finding of tuberculosis: a randomised controlled trial. Thorax..

[15] Menzies D, Benedetti A, Paydar A, Royce S, Madhukar P, Burman W (2009). Standardized treatment of active tuberculosis in patients with previous treatment and/or with mono-resistance to isoniazid: a systematic review and meta-analysis. PLoS Med..

[16] World Health Organization (2011). Guidelines for the programmatic management of drug-resistant tuberculosis – 2011 update.

[17] Fitzpatrick C, Floyd K (2012). A systematic review of the cost and cost effectiveness of treatment for multidrug-resistant tuberculosis. Pharmacoeconomics..

[18] Suárez PG, Floyd K, Portocarrero J, Alarcón E, Rapiti E, Ramos G (2002). Feasibility and cost-effectiveness of standardised second-line drug treatment for chronic tuberculosis patients: a national cohort study in Peru. Lancet..

[19] Pooran A, Pieterson E, Davids M, Theron G, Dheda K (2013). What is the cost of diagnosis and management of drug resistant tuberculosis in South Africa?. PLoS One..

[20] Kendall EA, Fofana MO, Dowdy DW (2015). Burden of transmitted multidrug resistance in epidemics of tuberculosis: a transmission modelling analysis. Lancet Respir Med..

[21] Faustini A, Hall AJ, Perucci CA (2006). Risk factors for multidrug resistant tuberculosis in Europe: a systematic review. Thorax..

[22] Espinal MA, Laserson K, Camacho M, Fusheng Z, Kim SJ, Tlali RE (2001). Determinants of drug-resistant tuberculosis: analysis of 11 countries. Int J Tuberc Lung Dis..

[23] Kliiman K, Altraja A (2009). Predictors of extensively drug-resistant pulmonary tuberculosis. Ann Intern Med..

[24] Boonsarngsuk V, Tansirichaiya K, Kiatboonsri S (2009). Thai drug-resistant tuberculosis predictive scores. Singap Med J..

[25] Martínez D, Heudebert G, Seas C, Henostroza G, Rodriguez M, Zamudio C (2010). Clinical prediction rule for stratifying risk of pulmonary multidrug-resistant tuberculosis. PLoS One..

[26] Vassall A, van Kampen S, Sohn H, Michael JS, John KR, den Boon S (2011). Rapid diagnosis of tuberculosis with the Xpert MTB/RIF assay in high burden countries: a cost-effectiveness analysis. PLoS Med..

[27] Pantoja A, Fitzpatrick C, Vassall A, Weyer K, Floyd K (2013). Xpert MTB/RIF for diagnosis of TB and drug-resistant TB: a cost and affordability analysis. Eur Respir J.

[28] Shin SS, Yagui M, Ascencios L, Yale G, Suarez C, Quispe N (2008). Scale-up of multidrug-resistant tuberculosis laboratory services, Peru. Emerg Infect Dis..

[29] Angeby KAK, Klintz L, Hoffner SE (2002). Rapid and inexpensive drug susceptibility testing of Mycobacterium tuberculosis with a nitrate reductase assay. J Clin Microbiol..

[30] El-Sayed Zaki M, Goda T. Rapid phenotypic assay of antimycobacterial susceptibility pattern by direct mycobacteria growth indicator tube and phage amplified biological assay compared to BACTEC 460 TB. Tuberculosis (Edinb). 2007;87:102–8. 10.1016/j.tube.2006.05.002 17035089

[31] World Health Organisation. WHO End TB Strategy. http://www.who.int/tb/post2015_strategy/en/. Accessed 18 January 2016.

[32] Miotto P, Piana F, Penati V, Canducci F, Migliori GB, Cirillo DM (2006). Use of genotype MTBDR assay for molecular detection of rifampin and isoniazid resistance in Mycobacterium tuberculosis clinical strains isolated in Italy. J Clin Microbiol..

